# Public health round-up

**DOI:** 10.2471/BLT.26.010226

**Published:** 2026-02-01

**Sudan: 1000 days of war**

On 9 January, the conflict in Sudan reached its 1000^th^ day. Nearly three years of continuous violence, severe access constraints and reduced funding have turned Sudan into the worst humanitarian crisis globally. An estimated 33.7 million people will need humanitarian aid this year. Sudan’s health system has been severely damaged by ongoing fighting, increasing attacks on health care, mass displacement, lack of essential medical supplies and shortages of health personnel.

**Figure:**
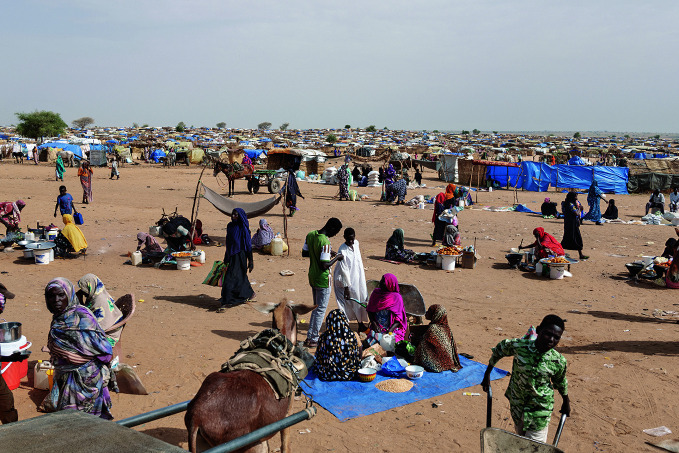
WHO/Nicolò Filippo Rosso

## Taxes on sugary drinks and alcohol

Sugary drinks and alcoholic beverages remain increasingly affordable in most countries due to low tax rates, contributing to rising rates of obesity, diabetes, heart disease, cancers and injuries.

In two global reports, *Global report on the use of alcohol taxes, 2025* and *Global report on the use of sugar-sweetened beverage taxes, 2025* the World Health Organization (WHO) urges governments to significantly increase taxes on these harmful products. 

Despite generating billions in profits, sugary drinks and alcohol remain largely under-taxed. WHO’s findings show that, while 116 countries tax sugary drinks and 167 tax alcohol, many taxes are low and fail to keep pace with inflation, making these products more affordable and accessible, and placing additional strain on health systems already burdened by preventable noncommunicable diseases and injuries. 

“Health taxes are one of the strongest tools we have for promoting health and preventing disease,” said Tedros Adhanom Ghebreyesus, WHO Director-General. “By increasing taxes on products like tobacco, sugary drinks and alcohol, governments can reduce harmful consumption and unlock funds for vital health services.”

https://bit.ly/4pQwkEv

## HIV clinical management 

WHO has released updated guidelines on human immunodeficiency virus (HIV) clinical management, offering revised recommendations on antiretroviral therapy, vertical HIV transmission prevention, and tuberculosis (TB) management. These updates reflect significant advancements in HIV treatment.

The new guidance confirms dolutegravir-based regimens as the preferred option for initial and subsequent HIV treatment, with updated recommendations for individuals whose current regimens are ineffective. WHO also supports the use of long-acting injectable treatments for those who face challenges adhering to daily oral regimens.

In preventing vertical HIV transmission, WHO emphasizes a person-centred approach, supporting exclusive breastfeeding for mothers with HIV while on effective antiretroviral therapy.

WHO’s updated TB prevention guidelines prioritize a 3-month course of isoniazid plus rifapentine for adults and adolescents living with HIV, addressing one of the leading causes of death in this group.

“These updated recommendations reflect WHO’s commitment to ensuring that people living with HIV benefit from the most effective, safe and practical treatment options available,” said Tereza Kasaeva, WHO director for HIV, TB, viral hepatitis and sexually transmitted infections. “By simplifying treatment, improving adherence and addressing persistent gaps in prevention, they will help countries strengthen HIV programmes and save lives.” 

https://bit.ly/4jSv9D6

## Global progress for trachoma 

The number of people requiring interventions against trachoma, the world’s leading infectious cause of blindness, has fallen below 100 million for the first time since global records began.

From an estimated 1.5 billion at risk in 2002, the number dropped to 97.1 million by November 2025, marking a 94% reduction. This achievement reflects decades of efforts by national health ministries, communities and international partners implementing the WHO-endorsed SAFE strategy (Surgery, Antibiotics, Facial cleanliness, and Environmental improvement).

“The reduction of the population requiring interventions against trachoma to below 100 million is testament to strong country leadership and consistent implementation of the SAFE strategy,” said Daniel Ngamije Madandi, WHO director for Malaria and neglected tropical diseases. “Progress across all trachoma-endemic WHO regions shows that SAFE is both effective and adaptable across contexts. WHO remains committed to supporting countries through the provision of technical assistance to achieve the global elimination of trachoma as a public health problem by 2030.”

Global progress has been supported by a diverse range of stakeholders, including non-governmental organizations, academic institutions and donors, many of which collaborate through the International Coalition for Trachoma Control, as well as the donation of more than 1.1 billion doses of azithromycin by Pfizer Inc. through the International Trachoma Initiative. These partnerships have enabled health ministries to distribute valuable donated medicines efficiently and effectively, while strengthening community health systems.

https://bit.ly/4r7MASH

## Human genomic technologies 

WHO has released a new global analysis on human genomics in clinical research, covering studies registered between 1990 and 2024. The report, *Human genomics technologies in clinical studies – the research landscape*, alongside an interactive dashboard, offers the most comprehensive overview to date of genomic technologies in clinical research, highlighting significant gaps in equity and inclusion.

The analysis reveals rapid growth in genomic studies, with over 6 500 studies globally and a sharp rise since 2010, driven by advancements in sequencing technologies and lower costs. Cancer and rare diseases dominate the landscape, but the report highlights stark inequities. Over 80% of studies are concentrated in high-income countries, with fewer than 5% in low- and middle-income countries, where limited research infrastructure often relegates these countries to secondary study sites.

The report also identifies demographic gaps, with over 75% of studies focusing on adults aged 18–64 years and minimal focus on children or older adults.

“Genomic technologies have extraordinary potential to transform health, however the disparities reflected in the report, unless strategically addressed, could reinforce existing inequities and limit the benefits of genomic science for populations who could benefit most,” said Meg Doherty, WHO director of the Department of Science for Health. “WHO will support efforts to ensure that genomic research reflects the diversity of global populations and public health needs.”

https://bit.ly/4baagkv

## Global waste crisis

A new WHO report, *Throwing away our health: the impacts of solid waste on human health – evidence, knowledge gaps and health sector responses*, warns that poorly managed solid waste is driving a global public health crisis and calls for urgent action to protect both people and the environment.

As municipal solid waste volumes grow at an unprecedented rate worldwide, many countries still lack the systems and resources to manage it safely. Improper waste management can release hazardous chemicals into air, water, soil and food, leading to contamination and health risks, particularly for vulnerable communities, including those near dumpsites, children, pregnant women and informal waste workers.

The report emphasizes that, when managed properly, waste can be turned into a resource, creating energy and green jobs. It advocates for a coordinated, multisectoral approach grounded in the waste hierarchy: prevention, reduction, reuse, recycling and safe disposal.

Key recommendations include reducing waste generation, expanding waste collection services in underserved communities and eliminating open dumping and burning. The health sector is urged to play a central role, from improving healthcare waste management to advocating for policies that protect public health.

“This report gives countries and health authorities a very practical agenda,” said Bruce Gordon, WHO unit head for Water, sanitation, hygiene, and health. “Health ministries can start now by ensuring safe management of health-care waste, developing strong occupational health programmes for waste workers, and working with municipalities to reduce health risks from solid waste by closing open dumps and burn sites and gradually improving towards safe services. These concrete steps save lives today and will make cities cleaner and healthier in the future.”

https://bit.ly/4pTPmKf

## Brazil eliminates mother-to-child HIV transmission

WHO has officially validated Brazil’s elimination of mother-to-child transmission of HIV, making it the most populous country in the Americas to achieve this significant milestone. This achievement reflects Brazil’s commitment to universal, free healthcare through its Unified Health System (SUS), supported by a strong primary health care system and respect for human rights.

“Eliminating mother-to-child transmission of HIV is a major public health achievement for any country, especially for a country as large and complex as Brazil,” said Tedros Adhanom Ghebreyesus, WHO Director-General. 

“This achievement shows that eliminating vertical transmission of HIV is possible when pregnant women know their HIV status, receive timely treatment, and have access to maternal health services and safe delivery,” said Jarbas Barbosa, PAHO Director. “It is also the result of the tireless dedication of thousands of health professionals, community health workers, and civil society organizations.”

https://bit.ly/4r3usJw

**Cover photo**

Empty playground by a lake in winter, Sweden

**Figure:**
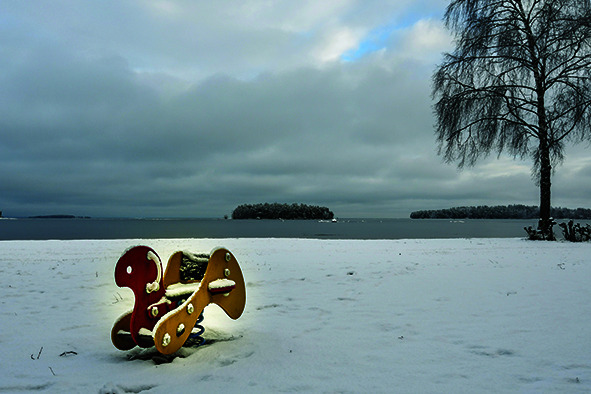
WHO/Malin Bring

**Looking ahead**

2–7 February. 158th session of the Executive Board, WHO Headquarters, Geneva. https://bit.ly/4jMvJ5e

23–26 February 2026. WHO consultation on the composition of influenza virus vaccines for use in the 2026-2027 northern hemisphere influenza season, Istanbul, Türkiye. https://bit.ly/4pRmPVD

